# The evaluation value of atherogenic index of plasma and high-sensitivity C-reactive protein for the degree of coronary artery lesion in premature coronary artery disease

**DOI:** 10.1186/s12872-024-04014-7

**Published:** 2024-08-06

**Authors:** Yuwei Song, Mengmeng Wang, Yijun Li, Yajun Lian

**Affiliations:** Department of General Practice, Heping Hospital Affiliated to Changzhi Medical College, 110 Yan’an South Road, Luzhou District, Changzhi, Shanxi Province 046000 China

**Keywords:** Premature coronary artery disease, Atherogenic index of plasma, High-sensitivity C-reactive protein, Degree of coronary artery lesion, Gensini score, Correlation, Receiver operating characteristic curve, Predictive value

## Abstract

**Background:**

Premature coronary artery disease (PCAD) is prevailing. We aimed to investigate the evaluation value of atherogenic index of plasma (AIP) and high-sensitivity C-reactive protein (hs-CRP) for the occurrence and severity of coronary artery lesion in PCAD patients.

**Methods:**

PCAD (PACD group)/non-PCAD (control group) patients were enrolled. The coronary artery lesion degree was evaluated using Gensini score (GS). PCAD patients were allocated into the low/medium/high GS groups, with general clinical baseline data analyzed. Plasma hs-CRP/AIP levels were compared in PCAD patients with different disease degree. Correlations between plasma hs-CRP/AIP with Gensini score, independent risk factors affecting the occurrence of PCAD, and the predictive value of hs-CRP/AIP/their combination for the occurrence and degree of PCAD were evaluated by Spearman correlation analysis/Logistic multivariate regression/receiver operating characteristic (ROC) curve. The differences in the area under the curve (AUC) were compared using MedCalc-Comparison of ROC curves.

**Results:**

Plasma hs-CRP/AIP levels in the PCAD group were increased. Plasma hs-CRP/AIP levels varied significantly among PCAD patients with different disease degree. Plasma hs-CRP/AIP levels were markedly positively correlated with the Gensini score. Smoking history/homocysteine/fasting blood-glucose/hs-CRP/AIP were all independent risk factors affecting PCAD occurrence. The AUC of hs-CRP and AIP combination predicting the occurrence of PCAD was 0.950 (90.80% sensitivity/93.33% specificity). hs-CRP/AIP combination assisted in predicting the disease degree in PCAD patients.

**Conclusions:**

AIP and hs-CRP are independent risk factors for the occurrence of PCAD, and their combination has high predictive value for PCAD occurrence and disease degree, which are both positively correlated with coronary artery lesion degree.

## Background

Coronary artery disease (CAD) represents the most prevailing type of atherosclerosis (AS)-induced organ lesions, referring to a clinical syndrome of myocardial hypoxia, necrosis, or ischemia triggered by coronary AS [1]. On the basis of the definition in relevant guidelines, premature coronary artery disease (PCAD) is defined as CAD in men < 55 years old and women < 65 years old, with AS as its basis of pathophysiology [2], and if the stenosis of vascular lumen reaches a certain degree, it will elicit cardiac insufficiency, as well as myocardial hypoxia and ischemia. Meanwhile, CAD atherosclerotic plaque has been revealed to largely consist of lipid, frequently leading to severe clinical phenotypes, which still causes a poor prognosis in the case of timely selection of standardized drug treatment and revascularization strategies, thus posing a heavy economic burden to families and society [3]. Consequently, early screening and intervention of PCAD are particularly prominent.

High-sensitivity C-reactive protein (hs-CRP) stands out as a liver-synthesized acute phase response protein, which is one of the most sensitive biomarkers of human non-specific inflammatory response, and can be recognized as an independent predictor for CAD onset and death [4]. hs-CRP plays critical roles in vascular disease occurrence and plaque rupture through several effects, including elevations in release of inflammatory factors, activation of the complement system, monocyte activation and chemotaxis, promotion on extracellular matrix remodeling, and lipid relevant effects, suggesting the fact that hs-CRP, as an inflammatory marker, directly stimulates arteriosclerosis formation and inflammation, and possesses the capability to change plaque structure and accelerate plaque rupture [5]. Notably, hs-CRP has also been confirmed to be produced in the monocyte macrophages and smooth muscle cells of local AS tissues [6]. Concurrently, hs-CRP plays a potential pathogenic role in the process of atherosclerotic plaque vulnerability, and high concentration of CRP in plasma is highly involved in the formation of thin fibrous cap [7], but its relevance in PCAD patients remains elusive. The atherogenic index of plasma (AIP), standing out as a sensitive indicator mirroring dyslipidemia, is intimately related to the arising of cardiovascular diseases [8]. The AIP value is the logarithm of the ratio of triglycerides (TG) to high-density lipoprotein cholesterol (HDL-C) [AIP = 1og (TG/HDL-C)] [9], with the calculation formula calibrated into log [(TG/HDL-C) × 100] to avoid negative values and ensure a normal distribution of the data. Accumulating evidence has demonstrated unequal lipid distribution levels in PCAD patients are, which means high TG and low HDL-C levels [10]. A previous study has also declared certain relevance between AIP with the onset of CAD and the degree of human AS [11]. Nevertheless, it remains unclear about the correlation between AIP and the degree of coronary artery lesion in PCAD patients. Accordingly, the study aims to delve into the correlations between hs-CRP and AIP with the degree of coronary artery lesion in PCAD patients, and further explore their evaluation value in disease degree of PCAD patients.

## Methods

### Ethics statement

The experiments were authorized by Heping hospital affiliated to Changzhi Medical College (Approval No. 2024-003). All procedures were strictly adhered to Declaration of Helsinki. All subjects involved were fully informed of the study objective and signed the informed consents before sampling.

### Study subjects

This study retrospectively enrolled 324 patients admitted to the cardiology department of Heping hospital affiliated to Changzhi Medical College with suspected CAD and receiving coronary angiography (CAG) examination from March 2021 to June 2023. Among them, 47 did not meet the inclusion criteria, 19 had incomplete data, and 20 were excluded following the exclusion criteria. Ultimately, 238 cases were included as the subjects of this study, with 163 PCAD patients as the disease group (PCAD group) and 75 non-PCAD patients as the control group. CAD was defined as the presence of infarctive stenosis in any major coronary artery, including the left main (LM), left anterior descending (LAD), left circumflex artery (LCX), and right coronary artery (RCA), or in the main branch of the vascular system, with a degree of stenosis reaching 50% of the lumen diameter [12].

### Inclusion and exclusion criteria

Inclusion criteria were as below: (1) male < 55 years old, female < 65 years old [13]; (2) the suspected CAD patients who underwent their first CAG examination; (3) no history of relevant medication use within 2 weeks before admission; (4) complete general clinical data and relevant examination data; (5) the PCAD patients with CAG results showing ≥ 50% degree of stenosis in at least 1 vessel.and.

Exclusion criteria were as below: (1) severe heart failure, acute coronary syndrome, or heart disease (New York Heart Association ≥ II); (2) severe kidney disorder or liver failure (serum creatinine > 3 mg/dL); (3) complication of thyroid disorder, malignant tumors or rheumatoid arthritis; (4) complication of severe acute infection or inflammatory disease; (5) had previously undergone intracoronary stent implantation or coronary artery bypass grafting; (6) undergoing immunosuppressive therapy; (7) had orally taken lipid-lowering drugs two weeks before admission.

### Data and sample collection

Clinical baseline data were collected from patients, comprising age, sex, body mass index (BMI), smoking history, drinking history, hypertension, diabetes, and family history of PCAD. A fully automated biochemical analyzer (Cobas c70, Roch, Germany) was employed for assessing fasting blood-glucose (FBG), plasma fibrinogen (FIB), homocysteine (Hcy), total cholesterol (TC), serum creatinine (Scr), TG, low-density lipoprotein cholesterol (LDL-C), and HDL-C. AIP was calculated as per the formula of AIP = log (TG/HDL-C), and calibrated by the calculation formula log [(TG/HDL-C) × 100] for preventing negative values and ensuring a normal distribution of the data. Based on the instrument manual, the determinations were strictly implemented on the same instrument by the same batch of personnel from the clinical laboratory. Three repetitions were guaranteed in each sample. Fasting venous blood (10 mL) were obtained from all subjects on the morning after admission, and placed in an anticoagulant-free general blood collection, 4 mL of which were allowed to stand for 30 min and centrifuged at 4 °C and 3000 r/min for 10 min, with the serum subpacked into eppendorf (EP) tubes, labelled, and stored in a refrigerator at -80 °C for standby. Whereafter, the remaining 6 mL of venous blood was put in an ethylenediamine tetraacetic acid anticoagulant tube, and was subjected to standing and centrifugation, followed by the plasma distribution into EP tubes, labelling, and storing at -80 °C for future use.

### Enzyme-linked immunosorbent assay (ELISA)

Level of plasma hs-CRP (JOTEK1805Hu) determination was conducted using an ELISA kit (Amyjet scientific, Wuhan, Hubei, China) strictly as per the manufacturer’s instructions.

### Assessment of coronary artery lesion degree

All study subjects underwent CAG after excluding relevant contraindications and signing the informed consent form. The degree of coronary artery lesion in PCAD patients was evaluated according to the Gensini scoring (GS) system developed by the American Heart Association [14]. The score of 1 point showed the degree of coronary artery stenosis ≤ 25%, 2 points manifested 26-50%, 4 points revealed 51-75%, 8 points elicited 76-90%, 16 points represented 91-99%, and 32 points showed 100%. Coefficient of lesion site were as follows: LM × 5; LAD or proximal-LCX × 2.5; mid-LAD × 1.5, distal-LAD × 1, mid to distal-LCX × 1; RCA × 1; small branch × 0.5. The score of each lesion was calculated as the degree of stenosis score multiplied by the lesion site score, and the GS score for each patient was the sum of all lesion scores. The angiographic findings were judged by two or more professional cardiologists. A higher GS represented higher degree of coronary artery lesion, with PCAD patients allocated into the low (≤ 21 points, 53 cases; mild lesions), medium (22–29 points, 64 cases; moderate lesions), and high (> 30 points, 46 cases; severe lesions) [15] GS groups based on the GS score.

### Statistical analysis

The data analysis and plotting were performed using SPSS 21.0 (IBM Corp. Armonk, NY, USA) and GraphPad Prism 8.0 (GraphPad Software, San Diego, CA, USA) statistical software. Whether the data were in a normal distribution was examined by Shapiro Wilk test (W test). The data of normal distribution were denoted as the mean ± standard deviation, and were compared between two groups by non-paired *t*-test and among several groups by one-way analysis of variance (ANOVA), followed by Tukey’s multiple comparison test. The data not in a normal distribution were represented by median values (minimum, maximum), followed by their comparisons between two groups utilizing Mann-Whitney test and comparisons among multiple groups utilizing Kruskal-Wallis H test. The categorical variables were analyzed using Fisher’s exact test. Spearman correlation analysis was conducted to evaluate the correlations between hs-CRP and AIP with the degree of coronary artery lesion. After all potential confounders were adjusted, logistic multivariate regression analysis was used to evaluate the independent risk factors affecting the occurrence of PCAD. The predictive value of hs-CRP, AIP, and their combination on the occurrence of PCAD and the degree of coronary artery lesion in patients was assessed by receiver operating characteristic (ROC) curves. The differences of area under the ROC curve (AUC) were analyzed and compared by MedCalc software. In a bilateral test, *P* < 0.05 was considered statistically significant for the difference.

## Results

### Clinical baseline data comparisons

The comparative analysis of the clinical baseline data of the two groupsuncovered no apparent difference in age, diabetes, family history of PCAD, TC, LDL-C between the two groups (all *P* > 0.05), whereas statistically obvious differences in terms of sex, BMI, smoking history, hypertension, drinking history, Hcy, FIB, TG, Scr, FBG, HDL-C, hs-CRP, and AIP (all *P* < 0.05, Table 1).

**Table 1:** Comparisons of general clinical baseline data between the PCAD group and the control group

Clinical Baseline Data	PCAD Group (*N* = 163)	Control Group (*N* = 75)	*P* Value
Age (years)	47.47 ± 6.17	48.60 ± 6.14	0.191
Sex [male, n (%)]	98 (60.12%)	34 (45.33%)	0.036
BMI (kg/m^2^)	25.73 ± 1.32	24.65 ± 1.27	< 0.001
Smoking History [n (%)]	76 (46.63%)	15 (20.00%)	< 0.001
Drinking history [n (%)]	51 (31.29%)	14 (18.67%)	0.044
Hypertension [n (%)]	93 (57.06%)	29 (38.67%)	0.012
Diabetes [n (%)]	39 (23.93%)	14 (18.67%)	0.406
PCAD Family History [n (%)]	24 (14.72%)	9 (12.00%)	0.688
FIB (g/L)	2.83 ± 0.52	2.66 ± 0.47	0.017
Hcy (µmol/L)	14.09 ± 3.07	10.65 ± 2.78	< 0.001
Scr (mmol/L)	72.06 ± 15.36	64.94 ± 14.23	0.001
TG (mmol/L)	1.82 ± 0.24	1.55 ± 0.23	< 0.001
TC (mmol/L)	4.46 ± 1.05	4.30 ± 0.97	0.25
LDL-C (mmol/L)	2.89 ± 0.73	2.75 ± 0.86	0.182
HDL-C (mmol/L)	0.95 ± 0.27	1.31 ± 0.41	< 0.001
FBG (mmol/L)	5.43 ± 1.26	4.69 ± 1.02	< 0.001
hs-CRP (ng/mL)	6.83 ± 2.46	3.54 ± 1.09	< 0.001
AIP	2.29 (2.02, 3.62)	2.09 (1.78, 3.62)	< 0.001

*Note* BMI: body mass index; FIB: fibrinogen; Hcy: homocysteine; Scr: serum creatinine; TG: triglyceride; TC: total cholesterol; LDL-C: low-density lipoprotein-cholesterol; HDL-C: high-density lipoprotein-cholesterol; FBG: fasting blood-sugar; hs-CRP: high-sensitivity C-reactive protein; AIP: atherogenic index of plasma; The measurement data of normal distribution were expressed as mean ± standard deviation, and independent sample *t*-test was utilized; Data not conforming to a normal distribution were represented by median values (minimum, maximum) and tested by Mann Whitney test; The categorical variables were compared and analyzed using Fisher’s exact test. *P* < 0.05 was indicative of statistical significance

### Comparisons of plasma hs-CRP and AIP levels in PCAD patients with different degree of coronary artery lesion

The plasma hs-CRP and AIP levels were compared among PCAD patients from the three groups, which disclosed higher plasma hs-CRP and AIP levels in the high GS group relative to the medium GS group (all *P* < 0.05), and higher plasma hs-CRP and AIP levels in the medium GS group versus the low GS group (all *P* < 0.05), suggesting that plasma hs-CRP and AIP levels gradually rose in PCAD patients along with the aggravation of degree of coronary artery lesion (Table 2).

**Table 2:** Comparisons of plasma hs-CRP and AIP levels in PCAD patients with different degree of coronary artery lesion

Indicators	Low GS group (*N* = 53)	Medium GS group (*N* = 64)	High GS group (*N* = 46)	*P*_a_	*P*_b_	*P*_c_
hs-CRP (ng/mL)	5.37 (0.23, 7.43)	6.38 (2.93, 9.18)	9.41 (7.36, 13.69)	0.008	< 0.001	< 0.001
AIP	2.19 (1.94, 2.45)	2.29 (1.92, 2.73)	2.43 (2.08, 3.71)	< 0.001	< 0.001	< 0.001

*Note* GS: Gensini score; hs-CRP: high-sensitivity C-reactive protein; AIP: atherogenic index of plasma; The measurement data were expressed by median values (minimum, maximum) and Mann Whitney test was used. *P* < 0.05 indicated a statistically significant difference. *P*_*a*_: Medium GS group compared with low GS group; *P*_*b*_: High GS group compared with low GS group; *P*_*c*_: the high GS group compared with the medium GS group

### Correlation analysis of plasma hs-CRP and AIP with Gensini score in PCAD patients

**Fig. 1:**
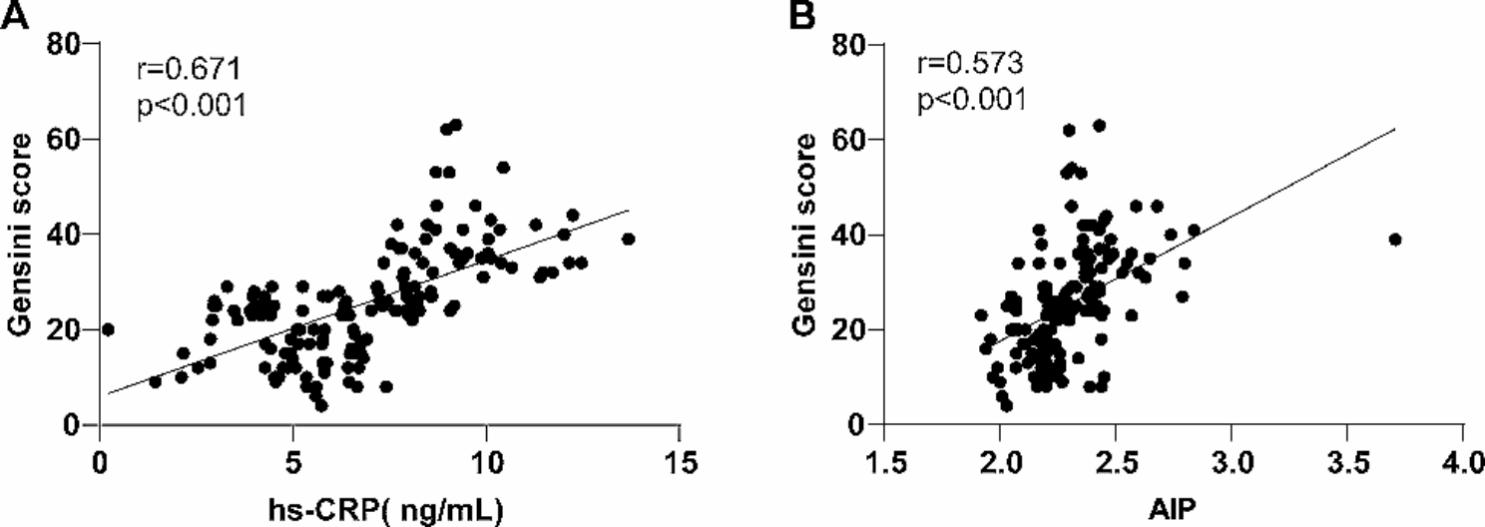
Correlation analysis of plasma hs-CRP and AIP with Gensini score in PCAD patients. (**A**-**B**) The correlations between plasma hs-CRP and AIP with Gensini score were analyzed using Spearman correlation analysis. Data in panels A-B were all analyzed by Spearman coefficient

To investigate the relationship between plasma hs-CRP and AIP levels with Gensini score in PCAD patients, we used Spearman correlation coefficients to analyze the correlations between plasma hs-CRP levels and AIP levels with Gensini score. Plasma hs-CRP and AIP levels both had a positive correlation with Gensini score (*r* = 0.671, 0.573, all *P* < 0.001) (Fig. 1A-B). Taken together, these outcomes suggested that as the hs-CRP and AIP levels rose, PCAD patients had gradually deteriorated degree of coronary artery lesion.

### High-expressed hs-CRP and AIP were independent risk factors for PCAD occurrence

With the occurrence of PCAD (0 = no, 1 = yes) as the dependent variable, we included sex, BMI, smoking history, drinking history, hypertension, Hcy, Scr, FIB, FBG, hs-CRP and AIP as the independent variables in the binary logistic multivariate regression analysis model. After all potential confounding factors were adjusted, the findings emanated that smoking history (*P* = 0.004, OR = 5.994, 95%CI: 1.780-20.188), Hcy (*P* = 0.001, OR = 1.516, 95%CI: 1.224–1.877), hs-CRP (*P* = 0.001, OR = 3.165, 95%CI: 2.021–4.956), FBG (*P* = 0.049, OR = 1.642, 95%CI: 1.001–2.692), and AIP (*P* = 0.002, OR = 54.527, 95%CI: 4.389-677.333) were independent risk factors influencing the occurrence of PCAD (Table 3).

**Table 3:** Logistic multivariate regression analysis of independent risk factors affecting the occurrence of PCAD

Variables	*P* value	OR	95%CI
Sex	0.16	2.379	0.710–7.969
BMI	0.109	1.824	0.874–3.807
Smoking History	0.004	5.994	1.780-20.188
Drinking history	0.474	1.587	0.448–5.621
Hypertension	0.487	1.485	0.487–4.523
FIB	0.585	0.694	0.187–2.572
Hcy	0.001	1.516	1.224–1.877
Scr	0.766	1.01	0.946–1.079
FBG	0.049	1.642	1.001–2.692
hs-CRP	0.001	3.165	2.021–4.956
AIP	0.002	54.527	4.389-677.333

### The predictive value of hs-CRP, AIP, and their combination in PCAD occurrence

**Fig. 2:**
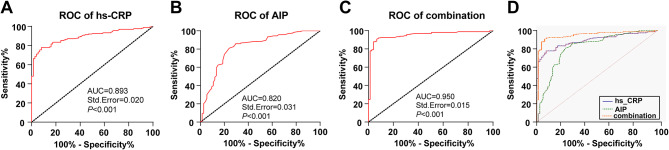
hs-CRP combined with AIP had high predictive value for PCAD occurrence. (**A**-**C**) ROC curve analysis was used to assess the predictive efficacy of plasma hs-CRP, AIP, and the combination of the two on PCAD occurrence; (**D**) The AUC difference between the two on PCAD occurrence was analyzed by MedCalc

Moreover, ROC curve was plotted to estimate the predictive value of plasma hs-CRP and AIP for the onset of PCAD. Subsequently, the AUC of plasma hs-CRP for predicting PCAD occurrence was 0.893 (95%CI: 0.853–0.932), the cutoff value was 4.745, with 77.91% sensitivity and 92.00% specificity, which indicated that plasma hs-CRP level > 4.745 (ng/mL) could aid in predicting PCAD occurrence. The AUC of AIP prediction for PCAD occurrence was 0.820 (95%CI: 0.760–0.880), the cutoff value was 2.175, with 80.98% sensitivity and 76.00% specificity, which identified that AIP value greater than 2.175 could assist in predicting PCAD occurrence. In addition, the outcomes disclosed that the combination of hs-CRP and AIP predicting the occurrence of PCAD had an AUC of 0.950 (95%CI: 0.920–0.980), a sensitivity of 90.80%, and a specificity of 93.33%. MedCalc analyses of the AUC differences implied that the combined prediction efficacy of hs-CRP and AIP surpassed their independent prediction efficacy for PCAD occurrence (all *P* < 0.01) (Table 4; Fig. 2A-D).

**Table 4:** Pairwise comparisons of ROC curves for hs-CRP, AIP, and their combination

	*P* Value
hs-CRP ~ AIP	0.078
hs-CRP ~ Combination	0.003
AIP ~ Combination	< 0.001

### The combination of hs-CRP and AIP helped predict coronary artery lesion degree in PCAD patients

**Fig. 3:**
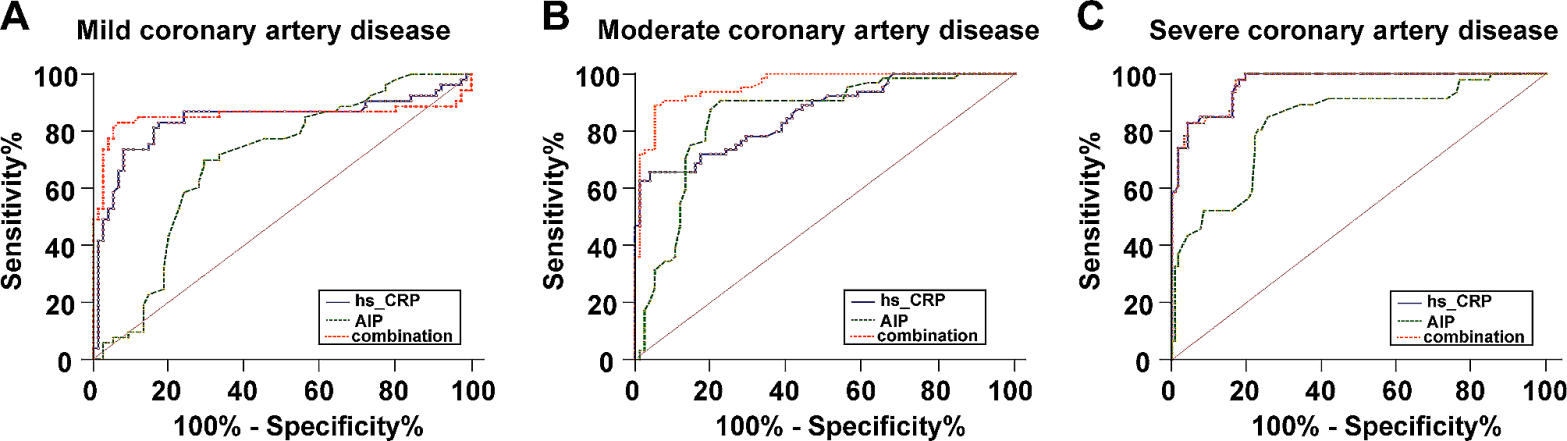
hs-CRP and AIP combination assisted in the prediction of coronary artery lesion degree in PCAD patients. (**A**-**C**) ROC curve was applied for appraising the predictive efficacy of plasma hs-CRP, AIP, and their combination in mild, moderate, and severe CAD in PCAD patients

As reflected by the ROC curve, the AUC values of plasma hs-CRP, AIP and their combination for mild CAD prediction were 0.839 (95%CI: 0.756–0.922, sensitivity of 83.02% and specificity of 82.67%), 0.694 (95%CI: 0.602–0.786, sensitivity of 69.81% and specificity of 70.67%), and 0.854 (95%CI: 0.767–0.942, sensitivity of 83.02% and specificity of 93.33%), those for moderate CAD prediction were 0.860 (95%CI: 0.799–0.921, 65.63% sensitivity, 96.00% specificity), 0.848 (95%CI: 0.781–0.916, 90.63% sensitivity, 77.33% specificity), and 0.961 (95%CI: 0.932–0.989, 89.06% sensitivity, 94.67% specificity), and those for severe CAD prediction were 0.966 (95%CI: 0.943–0.989, 100.00% sensitivity, 80.34% specificity), 0.828 (95%CI: 0.755-0.900, 84.78% sensitivity, 74.36% specificity), and 0.967 (95%CI: 0.944–0.989, 100.00% sensitivity, 80.34% specificity) (Table 5; Fig. 3A-C).

**Table 5:** Pairwise comparisons of ROC curves for hs-CRP, AIP, and their combination

	Mild CAD	Moderate CAD	Severe CAD
	*P* value	*P* value	*P* value
hs-CRP ~ AIP	0.012	0.821	< 0.001
hs-CRP ~ combination	0.358	< 0.001	0.81
AIP ~ combination	0.002	< 0.001	< 0.001

## Discussion

CAD represents the primary reason contributing to death in both the developed and developing countries [16]. AIP is recognized as an indicator of atherogenic dyslipidemia, which is remarkedly related to the progression of atherosclerotic cardiovascular disease [17]. Interestingly, cross-sectional studies have documented that higher AIP values may be relevant to CAD independently in grown-ups [11]. Meanwhile, hs-CRP can be considered as an independent predictor of CAD mortality, as well as incidence [4]. In light of this, our experimental findings highlighted that plasma hs-CRP and AIP were apparently augmented in patients with PCAD, and the levels were up-regulated dependently with the worsening of the degree of coronary artery lesion. Moreover, plasma hs-CRP and AIP were independent risk factors influencing the occurrence of PCAD, with their combination helping predict the occurrence of PCAD and the degree of CAD.

Inflammation have been shown to exert a significant influence on the early phases of atherosclerotic plaque formation [18], suggesting the possibility that inflammatory biomarkers may be utilized as latent tools for individualization of the risk assessment. For instance, CRP has been found to be among the most prevailingly known inflammatory biomarkers, exhibiting consistency in large prospective research as a risk factor of similar significance as traditional risk factors for cardiovascular diseases [19]. Furthermore, hs-CRP levels are proved to be increased rapidly among patients with acute idiopathic pericarditis [20]. Meanwhile, a previous study has evidenced that the AIP is correlated with the burden of AS [11]. With these support, our findings also revealed notably increased plasma hs-CRP and AIP levels in PCAD patients versus the controls. Significantly, GS serves as an effective tool for assessing the severity of CAD [21]. In early risk stratification, AIP may function as a simple alternative of residual risk [22]. Similarly, in research with the AIP as a continuous variable, higher AIP is indicative of higher probability of developing CAD [23]. Additionally, the 26 (32.5%) patients in hs-CRP low-risk group have a mean angiographic GS of 11.8+/-5.8, and 18 (22.5%) patients in moderate-risk group show a mean score of 28.9+/-7.9, and 36 (45%) patients in high-risk group exhibit a mean score of 78.7+/-41.0, suggesting that serum hs-CRP level present salient correlation with the severity of CAD as evaluated by angiographic GS [24]. GS has been confirmed to be associated with serum CRP (OR: 0.98, 95%CI: 1.05–1.35, *p* = 0.01) through a logistic regression analysis, with its level exhibiting a close relationship with the degree of CAD on the angiogram [25]. hs-CRP has a positive correlation with the GS, which is capable of indicating the severity of cardiovascular disease to some extent [26]. A study has demonstrated that as a conventional predictor of adverse cardiac events (MACE), hs-CRP represents a hepatocyte-synthesized acute-phase protein when the body is stimulated by tissue hypoxic injury or microbial invasion, which engages in the formation process of coronary atherosclerotic plaque, and is capable of reflecting post-percutaneous coronary intervention (PCI) treatment MACE and short-term prognosis of patients with early-onset acute myocardial infarction [27]. Also, some reports have manifested that hs-CRP, a readily detectable inflammatory marker, possesses the ability to assess the risk of CAD and the degree of stenosis in combination with other indicators, which is generally in agreement with the outcomes of this study [28]. It has been proven that the AIP is able to forecast the chronic total occlusion presence, as well as disease degree [29]. It is noteworthy that AIP is bound up with the presence and degree of acute coronary syndrome independently in young patients in a sex-dependent manner, which may be better than traditional lipid profiles [30]. Importantly, AIP has been revealed to be a greater predictor for cardiovascular disorders versus HDL-C or TG, which is of great significance for the prevention and therapeutic guidance of cardiovascular disorders if combined with conventional lipid measurements [31]. Furthermore, Kenan Toprak has elicited in his study on patients with non-ST-segment elevation myocardial infarction undergoing PCI treatment that AIP can act as a predictive biomarker for contrast-induced nephropathy, with potential advantages for the diagnosis of cardiovascular diseases and the prevention of the complications [32]. Importantly, elevation of hs-CRP level possesses the capacity to predict risk of all-cause, cardiovascular mortality independently in the common people [33]. Consequently, we assigned PCAD patients into the high-, low, and medium GS groups, and also exposed significant differences in plasma hs-CRP level and AIP level among PCAD patients of different degree of coronary artery lesion, with obviously positive correlations between plasma hs-CRP and AIP levels with GS. These evidence altogether confirmed that as hs-CRP and AIP levels increased, the disease severity of PCAD patients aggravated, and both of them had predictive value for CAD occurrence in PCAD patients, with their combination exhibiting more prolonged efficiency. Moreover, our findings highlighted that smoking history, Hcy, FBG, hs-CRP and AIP were independent risk factors influencing the occurrence of PCAD.

## Limitations of the study

This study was a single center retrospective study with a small sample size. Another limitation was that one of the major challenges of cytokine analysis was the availability of suitable analytical tools with high accuracy, specificity, precision, stability, linearity and analytical sensitivity. However, cytokine measurements in human blood have not been properly calibrated. It is critical to recognize the virtual impossibility of eliminating the effects of all potential confounding factors in experimental studies. Despite this challenge, a concerted effort was made to thoughtfully consider the inclusion and exclusion criteria for the trial in order to minimize its impact. Nevertheless, any unrecognized inflammation prior to the study, anti-inflammatory therapies applied by the patient (without the physician’s knowledge), or undiagnosed cancer and diabetes could prominently affect the tested cytokine levels. Additionally, we did not control for the factors such as dietary structure and race that might cause changes in AIP. Accordingly, attention should be paid to the potential role of patient demographics and lifestyle factors in modulating cytokine levels and their impact on PCAD. To better understand the relationship between cytokines and the extent of CAD, other variables can be included in the study, such as age, sex, physical activity levels, and dietary habits. For instance, Osadnik et al. have found that young healthy adults who have family history of PCAD show adverse dietary habits [34]. Significantly, a previous study has declared that people who have late bedtime is more likely to get PCAD [35]. Additionally, considering the genetic factors-caused variability of cytokine expression levels may provide a more detailed understanding of their role in PCAD pathology. As a consequence, our future direction is to fully incorporate the influential factors and expand the central sample size to more accurately classify and assess the cardiovascular risk in this specific population and to more effectively identify, manage, and treat those at high risk for PCAD.

## Conclusions

In summary, this study found that AIP and hs-CRP were independent risk factors for PCAD occurrence, and their combination had high predictive value for the occurrence of PCAD and the degree of coronary artery lesion, which could reduce the the rate of missed diagnosis of PCAD to some extent. hs-CRP and AIP will be reliable indicators in PCAD applications.

## Data Availability

The data that support the findings of this study are available from the corresponding author upon reasonable request.

## Abbreviations

CAD: coronary artery disease
PCAD: premature coronary artery disease
ROC: receiver operating characteristic
AS: atherosclerosis
hs-CRP: high-sensitivity C-reactive protein
AIP: atherogenic index of plasma
TG: triglycerides
HDL-C: high-density lipoprotein cholesterol
CAG: coronary angiography
CABG: coronary artery bypass grafting
BMI: body mass index
FIB: plasma fibrinogen
Hcy: homocysteine
Scr: serum creatinine
TC: total cholesterol
FPG: formamidopyrimidine-DNA glycosylase
LDL-C: low-density lipoprotein cholesterol
ELISA: Enzyme-linked immunosorbent assay
LMCA: left main coronary artery
LAD: left anterior descending artery
LCX: left peripheral coronary artery
RCA: right coronary artery
AUC: the area under ROC curve
CI: confidence interval
OR: odds ratio
